# Supplementary material for: High-Efficiency Reducing Strain for Producing Selenium Nanoparticles Isolated from Marine Sediment
Source: Int J Mol Sci. 2022 Oct 8;23(19):11953. doi: 10.3390/ijms231911953 (PMC9569793; doi:10.3390/ijms231911953)
Supplement: Supplementary file 1 [file ijms-23-11953-s001.zip › ijms-1929158-supplementary.pdf]

Table S1. Preliminary screening of sodium selenite reducing strains (plate scribing method).

Number	Strain Number	Colony Color Depth
1	12	++
2	8	+++
3	14	+++
4	33	+++++
5	21	++++
6	37	+
7	74	++
8	45	+++++
9	5	++
10	70	+
11	22	++
12	76	+++
13	71	++
14	13	++
15	6	+
16	24	++++
17	27	+++
18	28	++
19	40	++
20	2	+++
21	59	++
22	67	++++
23	25	+
24	73	+++
25	66	+
26	34	+++
27	53	+++
28	58	++++
29	72	+++++
30	36	+
31	11	-
32	64	-
33	38	-
34	75	-
35	55	-
36	68	-
37	62	-
38	56	-
39	15	-
40	26	-

"+" The number of it indicates the red depth.

"-" No red produced

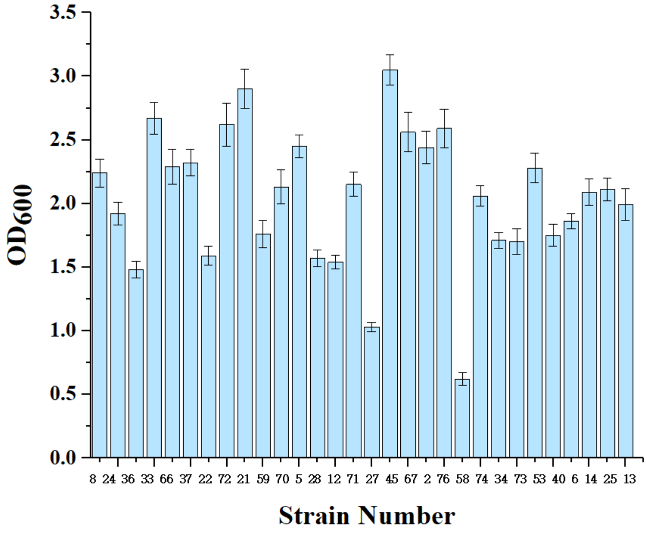

Figure S1. Re screening of sodium selenite reducing strains (liquid method).

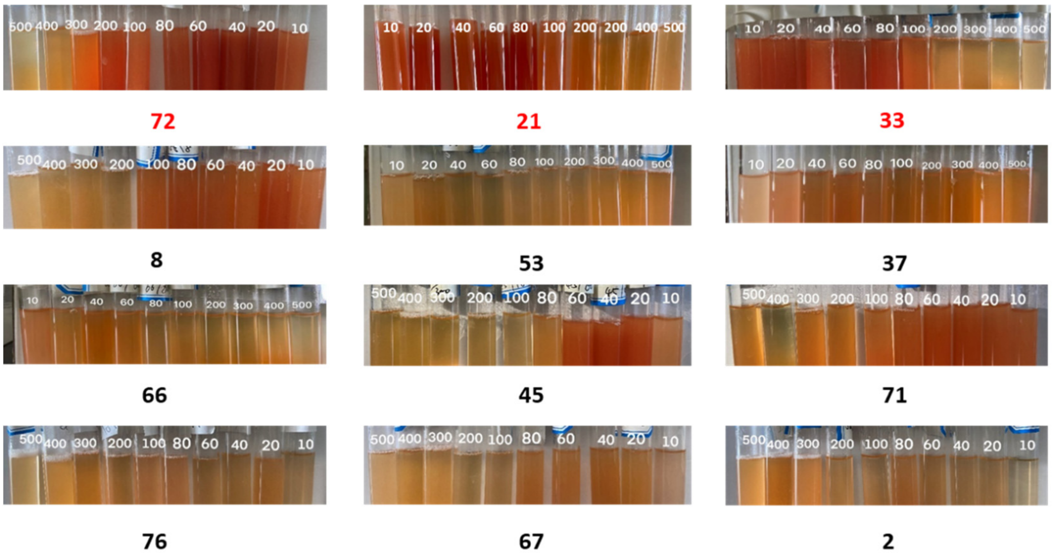

Figure S2. Reduction of 12 strains to different concentrations of sodium selenite.
